# Role of Point of Care Ultrasonography in Patients with COVID-19 Associated Acute Kidney Injury 

**DOI:** 10.24908/pocus.v7iKidney.15344

**Published:** 2022-02-01

**Authors:** Daniel W Ross, Zubair Hasan

**Affiliations:** 1 Division of Kidney Diseases and Hypertension, Donald and Barbara Zucker School of Medicine at Hofstra-Northwell; 2 Division of Pulmonary, Critical Care, and Sleep Medicine, Donald and Barbara Zucker School of Medicine at Hofstra-Northwell

**Keywords:** POCUS, acute kidney injury, COVID-19, point of care ultrasound

## Abstract

The severe acute respiratory virus covariate-2 (SARS CoV-2) that causes Corona Virus Disease 2019 (COVID-19) has affected more than 194 million people worldwide and has attributed to or caused more than 4 million deaths. Acute kidney injury (AKI) is a common complication of COVID-19. Point of care ultrasonography (POCUS) can be a useful tool for the nephrologist. POCUS can be used to elucidate the cause of kidney disease and then also help to manage volume status. Here, we review pearls and pitfalls of using POCUS to manage COVID-19 associated AKI with special attention to kidney, lung, and cardiac ultrasound.

## Introduction

The severe acute respiratory virus covariate-2 (SARS CoV-2) that causes COVID-19 has affected more than 194 million people worldwide and has attributed to or caused more than 4 million deaths as of July 26^th^, 2021 1. The reported incidence of acute kidney injury (AKI) among patients with COVID-19 vary. In a cohort of 5,449 hospitalized patients in the United States the incidence was reported as 37% 2. The outcomes among these patients is reported to be poor with 14% requiring dialysis and 35% ultimately dying. Intravascular volume excess frequently complicates acute kidney injury and has been shown to be a poor prognostic indicator in patients with respiratory failure and this remains true in those with COVID-19 3, 4. 

Point of care ultrasonography (POCUS) of the kidneys, lungs, and heart can be crucial to effectively diagnose and manage hospitalized patients with acute kidney injury and COVID-19. A trained nephrologist with a portable ultrasound can make important contributions without exposing additional staff like transporters or technicians. A recent review of the literature has found that most publications on the role of POCUS in COVID 19 have been limited to case series and opinions 5. However, some data do exist, and some data may be extrapolated from the general medical literature on POCUS. In order to demonstrate the utility of POCUS in managing COVID-19 associated AKI we will present the hospital course of a hypothetical patient that is based upon a real example. 

Mr. R is a 46-year-old man with no medical history who presents to the emergency room with difficulty breathing following 10 days of fever, diarrhea, and cough. He is found to have COVID-19 and a serum creatinine of 2.0 mg/dL (176.84 µmol / L). 

The first task for the nephrologist is to differentiate AKI from chronic kidney disease (CKD). Hirsch et al. reported that among patients with COVID-19 and AKI, baseline serum creatinine was known in only 15%. Ultrasound at the point of care can help to differentiate between AKI and CKD. The combined thickness of the cortex and medulla on ultrasound is known as the parenchymal thickness and in normal individuals is up to 1.5 cm 6. In CKD, the parenchyma will be less than 1.5 cm and the sinus fat will appear to be expanded. Additionally, the presence of simple bilateral cysts can indicate the presence of CKD 7. In this way, POCUS is a quick and effective tool for making this first, important diagnostic step. 

The next question for the nephrologist is whether Mr. R’s AKI is attributable to a reversible cause, like urinary obstruction. Classic epidemiological studies of AKI in hospitalized patients estimate that urinary obstruction accounts for 1% of cases of AKI in the hospital and rates among COVID-19 associated AKI are similar 8, 9. However, urinary obstruction remains an important diagnosis because it is more easily treatable. In the hands of a trained practitioner, POCUS carries a reasonable sensitivity to rule out hydronephrosis 10, 11, 12. 

Acute tubular necrosis (ATN) is the most common finding among patients with COVID-19 associated AKI 8. Even on biopsy series where other pathologic processes were found, all cases were complicated by ATN 13. Reports on the specificity of ultrasound for diagnosing ATN are outdated. Early reports have suggested that renal ultrasound and Doppler might help with prognostication, but unfortunately, in ATN, kidneys often appear normal 14, 15, 16. The prevailing opinion is that renal ultrasonography is of limited utility for differentiating ATN from other forms of AKI 17. It is notable that Doppler waveforms of the venous system are able to accurately identify AKI from venous congestion. The Venous Excess Ultrasound Grading System (VExUS) looks at inferior vena cava (IVC) size along with hepatic, portal, and intra-renal venous waveforms. A congested kidney by these metrics is strongly associated with the development of AKI after cardiac surgery 18.

In summary, urinary obstruction in COVID-19 associated AKI is rare and kidney ultrasonography is of limited utility for diagnosing ATN—the most common form of COVID-19 associated AKI. The most important information clinicians are likely to get from kidney POCUS is differentiating AKI from CKD in an otherwise undifferentiated patient with no known baseline kidney function. Acute kidney injury on CKD in hospitalized patients with COVID-19 is associated with a higher likelihood of requiring dialysis upon discharge 19. Identifying these patients with kidney POCUS can help nephrologists prognosticate and guide important clinical decisions. 

Despite best medical practices, including volume resuscitation, Mr. R gets intubated. He quickly becomes oliguric and his oxygen saturation is dropping. 

Is Mr. R’s oxygen saturation dropping because he is developing pulmonary edema from renal failure? Or is Mr. R’s oxygen saturation dropping because his disease is worsening, and his oliguria is due to days of insensible loss from fever, poor oral intake, and diarrhea? POCUS can be a valuable tool in these scenarios and nephrologists should understand how lung and cardiac ultrasound can be used to augment the traditional physical examination. 

## Lung Ultrasonography (LUS)

When an ultrasound wave meets a water-thickened interlobular septum, a linear reverberation artifact called a B-line juts away from the pleural surface 20. In cardiogenic pulmonary edema, these lines will arise from a smooth sliding pleura and will be homogenously distributed both between ribs interspaces and across both hemithoraces 20. There is an abundance of literature showing that B-lines on LUS correlate with extravascular lung water. In appropriate clinical setting extravascular lung water is associated with cardiac filling pressures. For example, one seminal study by Lichtenstein showed that B-line pattern (diffuse bilateral B-lines) correlates with an elevated pulmonary artery occlusion pressure 21. In patients with kidney failure on hemodialysis, B-lines are known to disappear in real time with ultrafiltration 22. In hemodialysis patients, B-lines inversely correlate with ejection fraction and directly correlate with mortality 23, 24. 

**Figure 1:**
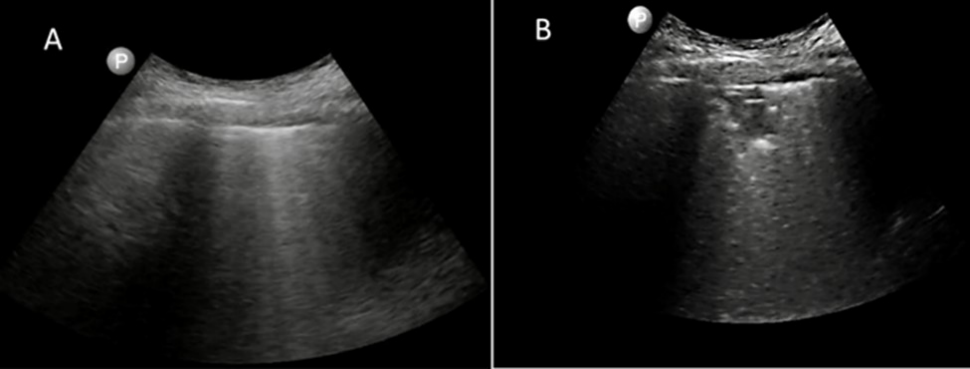
Lung ultrasound images taken using a curvilinear probe (C5 PhilipsLumify^TM^) in a patient admitted with COVID-19 pneumonia. Image of an interspace was saved in a six second loop. Both A and B are from the same interspace in the same loop. Image A seems to show B-lines originating from a smooth pleura and could easily be mistaken for pulmonary edema. Image B shows that during the respiratory cycle a subpleural consolidation with trace pleural effusion become evident. This case exemplifies why lung ultrasonography needs to be interpreted with caution when assessing volume status in patients with COVID-19.

Lung ultrasonography might be an attractive tool for evaluating extravascular lung water and thereby volume status in patients with COVID-19 associated AKI. Yet, there are potential pitfalls. It is critically important for the POCUS practitioner to be able to distinguish between lung ultrasound findings consistent with pulmonary edema and those findings consistent with COVID pneumonia. Figure 1 demonstrates typical lung ultrasound findings in COVID-19.

In a case series by Yasukawa in Washington D.C., 10 patients presenting to the emergency department with COVID-19 all had a diffusely irregular pleura, all the patients had areas of confluent B-lines and half also had subpleural consolidations 25. Subpleural consolidations can be particularly challenging for the novice POCUS practitioner. These consolidations appear as a subtle hypoechoic area directly underneath the pleura. If the ultrasound depth is set incorrectly, it can be easy to mistake these for the septal B-lines of early cardiogenic pulmonary edema.

A case series of 3 physicians with COVID-19 on home isolation showed a typical pattern in mild disease from an irregular pleura with scattered B-lines and subpleural consolidations that progressed to confluent B-lines on day 5 to 7 of illness followed by resolution back to A-line pattern 26. In a prospective study of 89 patients with severe COVID-19 disease, all patients had B-lines and these were confluent in 78.6%. Similarly, 78.6% of patients had pleural line irregularities in > 6 intercostal regions. Surviving patients were followed at 5 to 6 weeks post discharge and at that time B-lines and irregular pleura were seen in 15.6% 27. Two reviews that have looked at the reported cases of lung ultrasonographic findings in COVID-19 have come to similar conclusions. COVID-19 pneumonia is often located posteriorly and peripherally. Early findings include a thickened, irregular pleura which progresses to scattered B-lines with subpleural consolidations and then finally result in confluent B-lines. With clinical recovery, there is a return to normal aeration pattern (A-lines) 5, 28. In the early stages of COVID-19, B-lines might appear to have skip lesions with an irregular pleura making them easy to differentiate from pulmonary edema. However, in the more advanced stages of COVID-19 pneumonia, B-lines become confluent and without careful attention to the pleural line, the findings can be misconstrued. Therefore, it is important to use your high frequency linear probe to analyze the pleura when questions remain. In the appropriate setting and when interpreted correctly, lung ultrasonography can reliably predict COVID-19 in patients with respiratory failure 29. 

## Focused Echocardiography 

Given the high mortality associated with volume overload identifying volume status in patients with COVID-19 associated AKI is critical. Lung ultrasound findings might be unreliable or misleading. This is where using your standard four cardiac views are helpful. The parasternal long axis can be used to look at mitral valve excursion and overall left ventricular function. The parasternal short axis can be used to look for left ventricular contractility and wall motion abnormalities. The apical four chamber view can be used to look for left ventricular and right ventricular size and function comparison, as well as pericardial effusion. The subxiphoid can be used to look at the inferior vena cava (IVC). An early case series from Wuhan, China described three cases where focused cardiac echo was used in patients with COVID-19 to identify new onset global left ventricular dysfunction and acute myocardial infarction 30. Echocardiography is a versatile tool and can even be used when the patient is in the prone position 31. 

**Figure 2:**
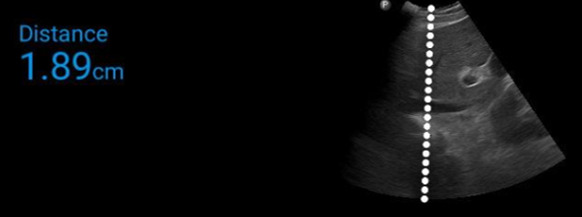
Inferior vena cava imaged with a curvilinear probe (C5 PhilipsLumify^TM^) from a subxiphoid approach.

Imaging of the inferior vena cava is may be useful in managing critically ill patients with AKI requiring dialysis and this remains true in COVD-19 32, 33. Shamsah et al. performed a prospective observational study of echocardiography in critically patients with COVID-19. In their analysis, they compared 77 patients admitted with COVID-19 who were not mechanically ventilated. They found that upon admission to the ICU, 80% of patients were noted to have an IVC < 2 cm and a collapsibility greater than 50%. The left ventricular ejection fraction of these patients was estimated to be 66% on average. These findings suggest that these patients were intravascular depleted when they entered the ICU. A collapsing IVC in a patient with COVID-19 associated AKI is shown in Figure 2. 

Despite best efforts, Mr. R's saturation drops below 80% on 100% FiO2 and therefore he requires extracorporeal membrane oxygenation (ECMO).

Patients with severe COVID-19 pneumonia and severe acute respiratory distress syndrome (ARDS) may remain hypoxemic despite best ventilator practices. These patients may require ECMO for life support if they remain hypoxemic. Depending on the type of failure the patient has, they may require veno-venous (VV) ECMO for primary respiratory failure, or veno-arterial (VA) ECMO for primary cardiac and respiratory failure. According to the worldwide Extracorporeal Life Support Organization registry, of the 2,132 cases of ECMO for COVID-19 reported thus far, 95% (2032) have been for VV-ECMO, reflecting the severe respiratory failure COVID-19 patients experience 34.There have been reports of COVID-19 related myocarditis, but these remain case reports and case series, so further studies are needed 35. As ECMO is being used more for COVID-19 support, it is important to recognize how management in these patients differs from other COVID-19 ICU patients. Traditional measurements of hemodynamics are unreliable with ECMO, and therefore POCUS becomes more important. 

To understand the role POCUS and transesophageal echocardiography plays in managing patients on ECMO, we must explain the different configurations of ECMO. In VV-ECMO, a large outflow/drainage cannula (21-25 Fr) is placed at the IVC/RA junction, typically via the femoral vein, along with an inflow cannula (17-21 Fr) into the SVC/RA junction. Sometimes femoral vein to femoral vein configurations are used and so it is important to know which configuration your patient has 36. These cannulas are inserted percutaneously or via cut down by the intensivist or cardiothoracic surgeon. Insertions are guided by fluoroscopy, or by echocardiography by visualizing the insertion of the guidewire into the IVC by utilizing a traditional subcostal view on ultrasound. VA-ECMO requires fluoroscopy and transesophageal echocardiography for insertion, with the inflow cannula sitting in the femoral artery at the level of the common iliac artery. 

POCUS is helpful in managing ECMO patients purely from a circuitry standpoint. For both VV- and VA-ECMO patients, POCUS helps identify atrial and ventricular size and function, valvular regurgitation, thrombus, the presence of pericardial effusion, and, most importantly, cannula position 37. A mispositioned ECMO cannula, either the drainage or inflow cannula, may result in sub-optimal ECMO function, and ultimately, patient hypoxemia and hypoxia 38. While on ECMO, special consideration must be given to the patient’s volume status, as it can affect the flows on the ECMO circuit. 

Nephrologists who use POCUS need to be aware of the limitations of interpreting IVC size and variability in patients on ECMO. The drainage cannula often sits in the IVC. This catheter can be anywhere from 21-25 Fr, meaning as much as 8.33 mm in diameter. Drainage cannulas are typically multi-stage cannulas, meaning they are perforated on the edges to allow for maximal drainage of the IVC. Therefore, the IVC must remain larger than the cannula diameter in order to ensure continuous, uninterrupted flow for the ECMO circuit 37. The IVC and drainage cannula can be seen in the subcostal view using POCUS. If the IVC appears to be collapsed around the cannula, the nephrologist and intensivist must exercise caution in further fluid removal, and in fact may need to give the patient fluid, in order to prevent “chattering” on the ECMO circuit, resulting in fluctuating flows and ultimately, hypoxemia. 

Sometimes, the cannulas may not be visualized using transthoracic echocardiography. These patients may need transesophageal echocardiography (TEE) to see if the cannulas are positioned appropriately. Transesophageal echocardiography can also be used to elucidate the cause of hypoxemia. Both trans-thoracic and trans-esophageal echocardiograms can be used to identify a right to left intra-cardiac shunt, such as due to a patent foramen ovale, that might explain a patient's hypoxemia. Transesophageal lung ultrasound (TELUS) is increasingly recognized as a tool to diagnose hypoxemia in patients who are too critically ill to move to a CT scanner and in whom other traditional imaging modalities are not practical or safe 38.TELUS can show lung consolidations at the bases in obese patients and whether there are A-lines, B-lines, irregular pleura, or pleural effusions. TEE can identify a thrombus in the pulmonary artery, a dilated right ventricle, or impaired left ventricular contractility^38^ TELUS can also help determine whether the patient’s hypoxemia can improve with recruitment maneuvers, or if there is a volume issue at play. 

While on ECMO, Mr. R required intermittent boluses of colloid due to fluctuating ECMO flows and an IVC collapsed around his drainage cannula, even though he clinically appeared euvolemic. POCUS showed improved aeration of his lung bases and anterior lung fields. He was able to be weaned off VV-ECMO. His IVC appeared dilated with no respiratory variation post-VV-ECMO, and fluid was restricted. His acute kidney injury resolved, and urine output improved. 

## Conclusion

Point of care ultrasonography has a variety of uses for the nephrologist evaluating COVID-19 associated AKI (Table 1). A nephrologist well trained in ultrasound can minimize exposure from additional staff such as technicians and patient transporters that are required for formal radiology studies. Kidney ultrasound can help identify obstruction and CKD. Many patients during the pandemic arrive with no known baseline kidney function. Chronic kidney disease is associated with a higher mortality and higher likelihood of requiring dialysis upon discharge. Therefore, POCUS can give the nephrologist important prognostic information. Ultrasound-based volume assessment is important in managing hypervolemia. Lung ultrasonography needs to be interpreted with caution because B-lines often arise from an irregular pleura due to the underlying pulmonary process, and can also be seen with subpleural consolidations. When patients require ECMO, TEE and TELUS can be useful for evaluating volume status. As COVID-19 continues to spread across the globe, nephrologists should become more informed about how POCUS can improve the care of their patients. 

**Table 1:** Point of Care Ultrasonography in COVID-19 Associated Acute Kidney Injury -- Key Points

Kidney and Bladder Ultrasound	· Urinary obstruction is an uncommon but reversible cause of AKI · Many patients have no known baseline creatinine and so POCUS is useful to identify CKD.
Lung Ultrasonography	· Confluent B-lines are common · Irregular pleura is common · Sub-pleural consolidations are common · Careful attention to the pleural line is needed for accurate interpretation
Echocardiography	· Many patients have collapsible IVC and hyperdynamic LV prior to intubation · When patients are on ECMO, TTE and TEE is useful for evaluating lungs and cardiac chambers, as well as cannula position. It can inform when not to remove fluid because of ECMO considerations.

## Disclosures

The authors have no conflicts of interest to disclose. 
